# The efficacy of manual therapy for chronic obstructive pulmonary disease: A systematic review

**DOI:** 10.1371/journal.pone.0251291

**Published:** 2021-05-18

**Authors:** Ji-Ae Roh, Kwan-Il Kim, Hee-Jae Jung

**Affiliations:** 1 Department of Clinical Korean Medicine, Graduate School of Kyung Hee University, Seoul, Republic of Korea; 2 Division of Allergy, Department of Internal Medicine, Immune and Respiratory System, College of Korean Medicine, Kyung Hee University, Seoul, Republic of Korea; University of Maryland School of Medicine, UNITED STATES

## Abstract

**Background:**

Manual therapy (MT) can be beneficial in the management of chronic obstructive pulmonary disease (COPD). However, evidence of the efficacy of MT for COPD is not clear. Therefore, we aimed to review the effects of MT, including Chuna, in people diagnosed with COPD.

**Methods:**

MEDLINE via PubMed, EMBASE, The Cochrane Central Register of Controlled Trials (CENTRAL), China National Knowledge Database (CNKI), KoreaMed, Korean Medical Database (KMbase), and Oriental Medicine Advanced Searching Integrated System (OASIS) were searched. Randomized controlled trials (RCTs) and crossover RCTs were included. The main inclusion criteria were COPD diagnosis (forced expiratory volume in the first second [FEV_1_]/forced vital capacity [FVC] < 0.70). The primary outcomes were lung function and exercise capacity. The secondary outcomes were symptoms, quality of life (QoL), and adverse event (AE)s. Studies reporting one or both of the primary outcomes were included. The Cochrane RoB 2.0 tool was used to assess the risk of bias. Data synthesis and analysis were conducted according to the trial design.

**Results:**

Of the 2564 searched articles, 13 studies were included. For the primary outcomes, the effect of MT on pulmonary function and exercise capacity in COPD was partly significant but could not be confirmed due to the limited number of studies included in the subgroups. For the secondary outcomes, no definitive evidence regarding the improvement of symptoms and QoL was found, and some minor adverse effects were reported.

**Conclusions:**

There is insufficient evidence to support the role of MT in the management of COPD. High-quality studies are needed to thoroughly evaluate the effect of MT on COPD.

## Introduction

Chronic obstructive pulmonary disease (COPD), characterized by persistent respiratory symptoms and airflow limitation caused by airway and alveolar pathologies, is a complex condition with various patterns of symptoms, progression, and associated comorbidities [1, 2]. The Global Burden of Disease Study estimated that there are approximately 174 million patients with COPD [3]. Moreover, 3.2 million patients worldwide died in 2015 due to COPD [4].

Patients with chronic respiratory diseases experience dyspnea, fatigue, and exercise intolerance [5, 6]. These conditions are associated with a reduced quality of life (QoL) due to a decrease in physical activity levels [7–10], and the clinical manifestations do not improve with appropriate pharmacological therapy [11, 12]. Patients with COPD not only present with respiratory diseases but also extra-pulmonary manifestations, muscle weakness, and medical and mental comorbidities [13, 14]. Therefore, non-pharmacological interventions to improve the QoL of patients with COPD are important [15, 16], and pulmonary rehabilitation (PR) is recommended for these patients [17].

PR [5], which has been shown to improve QoL, is a multidimensional therapy that encompasses education, physical exercise, and mental assistance. Physical exercise, as the primary intervention, includes active exercises such as walking and stair exercises. Some researchers recommend Manual therapy (MT) as an additional treatment option in association with other interventions, such as physical exercise [18], and MT that targets the respiratory muscles would be particularly beneficial for patients with COPD to develop muscle strength and maintain muscle movement [19].

Although several systematic reviews (SRs) assessing the efficacy of MT have been published [20–23], it is difficult to reach a conclusion due to the contradictory results. Heneghan (2012) [20] concluded that MT relieves dyspnea and improves overall health conditions but reported only minimal improvement in pulmonary function. Galletti et al.’s study [22] suggested a link between MT and an improvement in physical ability level; however, no clinically significant improvements in QoL or pulmonary function were observed. Nevertheless, the existing systemic reviews have limitations since they included research on MTs published only in English and excluded non-English research.

In Asian countries, a technique called Chuna adds the traditional concept of meridian massage to MT [24]. In particular, Korea has developed a unique form of Chuna by combining it with traditional Korean medicine [25, 26]. The effects of Chuna on musculoskeletal pain relief [27, 28], as well as its efficacy in the treatment of internal diseases [29–33], have been reported. However, there has been no research on its effects in COPD treatment. Thus, this study intended to elucidate the efficacy of MT, including Chuna (which is used in Korea and China), in the COPD population.

## Materials and methods

This study was conducted according to the guidelines in the Cochrane Handbook for Systematic Reviews of Interventions [34] and reported according to the Preferred Reporting Items for Systematic reviews and Meta-Analyses guidelines (PRISMA) [35] (S1 File). The protocol for this SR was registered in the international prospective register of SRs [36] with the identifier CRD42019141150. The review process was pre-specified in a published protocol to prevent reporting and researcher bias [37]. All steps were performed independently by two researchers, and discrepancies were resolved by another researcher.

### Eligibility criteria

The eligibility criteria for this review were based on the participants, intervention, comparison, outcomes, and study design (PICOS) framework of the PRISMA guidelines [38].

#### Study type

All randomized controlled trials (RCTs) and crossover RCTs were included. The following types of articles were excluded: non-RCTs, quasi-RCTs, observational studies, case reports, case series, reviews, and studies with animal experiments.

#### Participants

Human participants aged over 18 years diagnosed with COPD were included regardless of sex, race, disease stage, or exacerbation history; additionally, they did not have to discontinue conventional therapy (CT) (tablets, inhalational agents) during the clinical trial. The Global Initiative for Chronic Obstructive Lung Disease (GOLD) report was used as a diagnostic criterion. If the diagnostic criterion in the GOLD report was not specified, populations with a history of COPD and lung function of forced expiratory volume in the first second (FEV_1_)/forced vital capacity (FVC) <0.70 were included in the review. The following participants were excluded from the review: subjects with comorbidities that could affect the respiratory system, such as lung cancer, or other respiratory diseases such as asthma.

#### Intervention

Studies on any type of MT were included, such as mobilization, spinal manipulative therapy, massage, and other techniques wherein the practitioners therapeutically maneuvered the patient’s body using their hands [39]. In addition, various techniques used by specialists in osteopathy, chiropractic care, Chuna, and techniques used by other healthcare providers were included.

MTs alone or in combination with other treatments were eligible; however, MT had to be the main intervention for outcome measurements. Studies on exercise therapy, acupressure, reflexology, home-based self-treatments, voluntary stretching, and therapies performed by non-specialists were excluded.

#### Comparison

Sham treatments, non-therapeutic touch, no treatment, routine PR, and treatments with medications alone were included for comparison. MT was excluded from the comparison.

#### Outcomes

Studies that reported any of the primary outcomes were eligible for inclusion. Secondary outcomes, if reported, were collected from the included studies.

*1*. *Primary outcome*. Two objective measurements were used for primary outcomes.

(1) Pulmonary function test (PFT): Six pulmonary function parameters (FEV_1_, FVC, FEV_1_/FVC, vital capacity [VC], residual volume [RV], and total lung capacity [TLC]) were included in the review. Spirometry is a means of diagnosing and evaluating COPD by measuring airway obstruction [17]. RV and TLC also represent hyperinflation, a major feature of COPD [40]. When at least one item of static and/or dynamic lung volume was reported, it was used to evaluate lung function.

(2) Exercise capacity: The six-minute walk distance (6MWD) test is frequently used in the COPD population to measure their functional exercise performance [41, 42]. It measures the time spent walking a distance of 30 m (100 ft) according to the standard guidelines of the American Thoracic Society [43]. This test provides information regarding performance of activities of daily living and mortality in patients with COPD [44].

*2*. *Secondary outcomes*. (1) Symptoms: All COPD symptoms described in the retrieved studies were extracted if reported. The modified Medical Research Council (mMRC) dyspnea scale, Borg scales, patient-reported measures, VAS (visual analogue scale) for dyspnea, and other standards were eligible [17].

(2) QoL: Tools such as the “COPD Assessment Test” (CAT) and “St. George Respiratory Questionnaire” (SGRQ), which are useful for assessing QoL, were eligible for inclusion in the review [17, 45]. If this information was reported, it was also extracted.

(3) Adverse event (AE): An AE is an undesirable and unintended sign, symptom, or disease that does not necessarily have a cause-and-effect relationship with the intervention evaluated in a clinical trial (e.g., soreness in muscles, increased pain, and stiffness) [46–48]. We extracted data on AEs whenever they were reported in the included studies.

### Database and search strategies

#### Electronic searches and other sources

This study was performed based on a previously described method [37]. Searches were conducted independently in online electronic databases, including MEDLINE via PubMed, EMBASE, the Cochrane Central Register of Controlled Trials (CENTRAL), and the China National Knowledge Infrastructure database, to retrieve research relevant to the use of MT in COPD. Additionally, the following three Korean medical databases were searched: KoreaMed, Korean Medical Database (KMbase), and Oriental Medicine Advanced Searching Integrated System. The reference lists of the retrieved articles and relevant SRs were searched manually. Ongoing RCT registers, such as Clinicaltrials.gov and the World Health Organization International Clinical Trials Registry Platform (WHO ICTRP), were also reviewed. The study authors were contacted to confirm any unconfirmed data whenever possible and necessary.

#### Search strategy

The method used in this study has been previously described [37]. Articles were searched using Related Medical Subject Heading terms and synonyms in various combinations in each database from inception through January 31, 2021, without language restrictions. The search strategies consisted of relevant disease- and intervention-level word combinations. The terms relevant to the disease included “COPD,” “emphysema,” and “bronchitis.” The terms relevant to interventions included “manual therapy,” “manipulation,” “mobilization,” “massage,” and specific words for treatment modalities such as “physiotherapy,” “osteopathy,” “chiropractic,” and “Chuna (Tuina).” The detailed process is shown in the S2 File. Only disease- and intervention-level words were used as electronic search terms. After searching the databases, two researchers evaluated whether the studies should be excluded according to the eligibility criteria of the review.

### Study selection

According to the pre-defined PICOS criteria, two reviewers (JAR and KIK) independently assessed the titles and abstracts of the search results. With the same set of inclusion criteria, the full text of the articles was reviewed for further inclusion by the two reviewers separately. All included studies were uploaded to EndNote X9.3.3, (Clarivate Analytics, Philadelphia, PA) for bibliographic management and review. Any disagreement was resolved by discussion with a third researcher (HJJ).

### Data extraction and management

A standard extraction form (a pre-designed Excel file) was used for data extraction by the two reviewers (JAR and KIK). Detailed raw data, such as publication year, first author, country, title, study design, group allocation, method of randomization, number of groups, blinding procedure, number of withdrawals and dropouts, inclusion/exclusion criteria, diagnostic criteria for COPD, number of participants, patient’s age, sex, COPD stage, patterns of symptoms based on traditional Korean medicine or traditional Chinese medicine (syndrome differentiation/pattern identification), interventions (the type of the intervention, number of treatments, duration of individual treatment, total number of treatments, type of practitioner), comparators, outcomes (primary and secondary outcomes), and AEs, were extracted.

### Assessment of risk of bias

The Cochrane RoB 2.0 tool was used to perform a literature analysis for the SR and meta-analysis of RCTs. The current version of RoB was separately used to assess the risk of bias in individually randomized parallel-group trials (2019), and the RoB 2.0 tool was used to assess the risk of bias in individually randomized crossover trials, wherein each trial was categorized into the one of the three groups: (1) high risk of bias; (2) some concerns; and (3) low risk of bias [49, 50].

The risk of bias consists of the following five domains: (1) bias arising from the randomization process, (2) bias due to deviations from the intended interventions, (3) bias due to missing outcome data, (4) bias in the measurement of the outcome, and (5) bias in the selection of the reported result. These five evaluation domains differed from the seven domains stated in our protocol [37].

Two reviewers (JAR and KIK) independently evaluated the risk of bias in the included studies, in accordance with the tool’s recommendations. The risk of potential biases was considered for studies in which data were missing or the process of analysis was unclear. Disagreements were resolved by consulting with another researcher (HJJ).

### Data synthesis and analysis

In addition to the previously described protocol [37], we further attempted to analyze the RCTs according to their research designs in this review. The analysis protocol was divided into four categories, as follows:

1. MT *versus* Sham
2. MT + CT *versus* CT alone
3. MT + PR *versus* Sham + PR
4. MT + PR *versus* PR alone

Heterogeneity was assessed using the I^2^ test, with a significance level of *P* <0.1. An I^2^ value of ≥50% was considered to be a substantial inconsistency according to the Cochrane handbook of systematic review of interventions (version 6.2 chapter 10.10.2) [51]. Fixed-effects modeling was applied to data with substantial homogeneity (I^2^ value <50%), and random-effects modeling was applied to data with heterogeneity (I^2^ value ≥50%, *P*-value <0.10).

For studies with missing data or unclear methodology, the authors were contacted via email to clarify the information. If there was no reply or insufficient data after contact, only the available data were used for analysis.

To consider heterogeneities other than statistical heterogeneity that can influence the results, rather than pooling all studies, we pooled a group of studies that had the same trial design and outcomes into a meta-analysis. Multi-arm RCTs were excluded from the meta-analysis. However, whenever two subgroups were reported for an intervention, we combined the two reported subgroups into a single group and pooled the mean (or mean change) and standard deviation (SD) of the combined group into the meta-analysis [52]. While the RoB results did not influence the pooling in the meta-analysis, they influenced the interpretation of the results.

The review manager software (RevMan, Version 5.4.1 for Windows; Copenhagen, The Nordic Cochrane Center, The Cochrane Collaboration, updated in September 2020) was used for data analysis. If the data were adequately homogeneous for analysis, a meta-analysis was performed using fixed- or random-effects models. Random-effects models were used to consider the heterogeneity between interventions in individual clinical research. The mean difference (MD), confidence interval (CI), and *P* values were used for analysis. We used the MD and SD of post-intervention values in the meta-analysis. If necessary, mean change scores and SD obtained pre- and post-intervention were used [52]. Weighted MDs with 95% CIs were calculated for continuous data obtained using the same measurement scale. In case measurements were made on a different scale, a standardized MD was used. Odds ratio, risk ratios with 95% CIs, and *P* values were used for dichotomous outcomes. When quantitative synthesis was not appropriate due to heterogeneity, we summarized the study characteristics and outcome measures and conducted a narrative synthesis. Since only a few studies with the same trial design were included in the meta-analysis, analyses with forest plots were not possible for all studies. Moreover, funnel plot, subgroup, and sensitivity analyses could not be performed as planned.

#### Grading the quality of evidence

The levels of evidence for outcomes and recommendation strengths were assessed according to the Grading of Recommendations, Assessment, Development, and Evaluation [53].

## Results

### Description of the included studies

**Fig 1:**
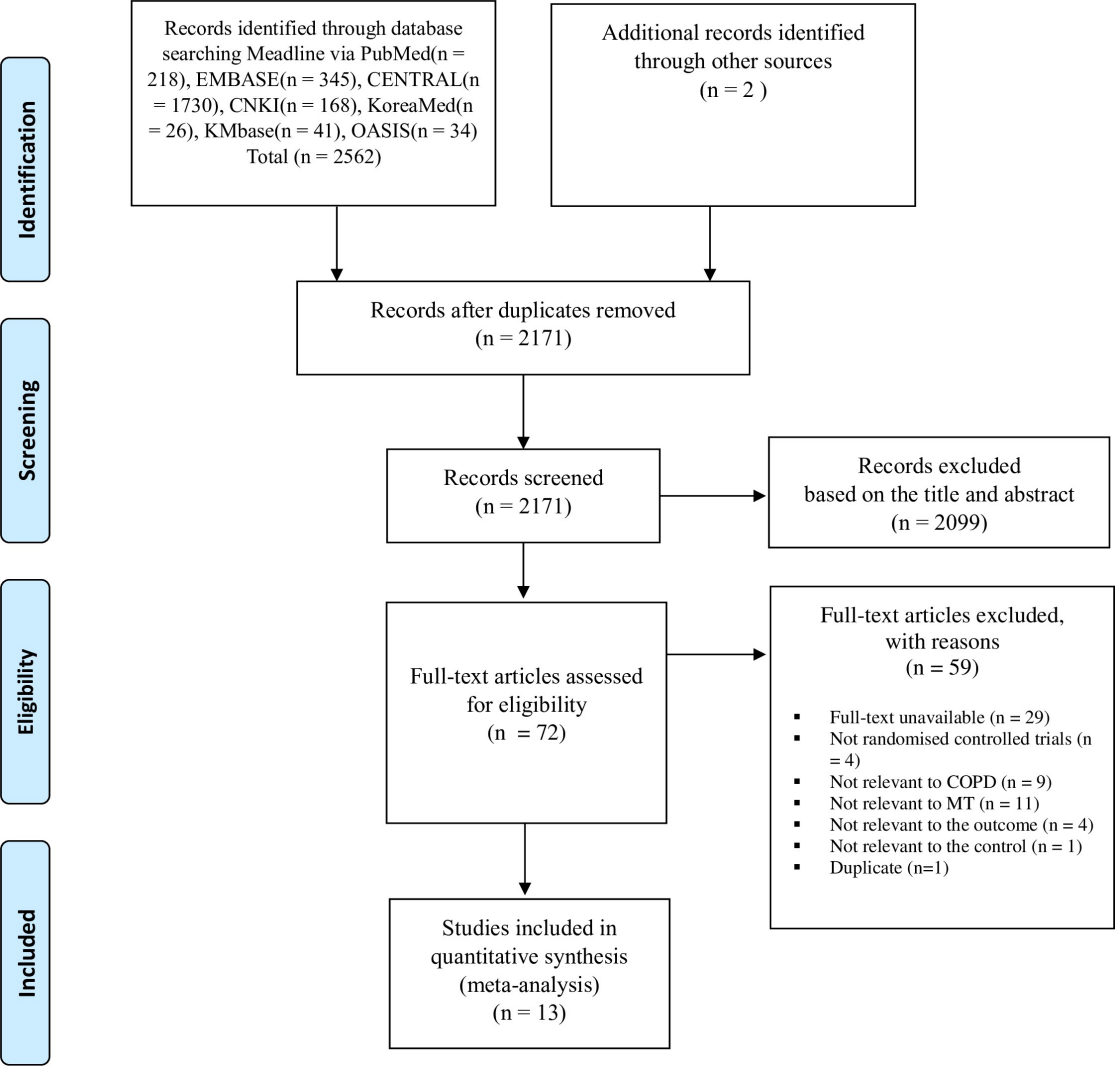
Flowchart for the identification and screening of eligible studies.

A total of 2562 articles were obtained from the initial search of seven databases, and two studies were additionally obtained from the bibliographic information of the retrieved articles. After excluding 393 duplicates from the 2564 articles, the titles and abstracts of 2171 articles were screened for eligibility. Further, 2099 articles were discarded because of the issues related to participants, interventions, or study type. After complete review, 59 articles were further excluded from the remaining 72 articles. Finally, among the 2564 retrieved articles, we included 13 RCTs that satisfied the eligibility criteria. All included RCTs [54–66] were used for qualitative analysis, and seven studies [57–60, 63, 64, 66] were included for additional quantitative analysis. A flow chart is presented in Fig 1.

The general characteristics of the included studies are summarized in Table 1. All 13 included studies were published in journal articles. Among the 13 included RCTs, 3 were crossover RCTs [55–57]. The year of publication of the included articles ranged from 1975 to 2019. Three of these studies were conducted in the United States [54, 55, 66]; two in Australia [57, 63]; two in Brazil [60, 64]; two in Italy [58, 61]; two in Poland [56, 59]; and one each in China [65] and Pakistan [62].

**Table 1:** Characteristics of the included studies.

Author (year) Country	Design	Participants	Treatment duration; Number of treatments	Treatment group (N *completed)	Comparator (N *completed)	Intervention	Outcomes
Noll et al. (2008) [54] US	RCT	Stable elderly COPD GOLD stage: unknown	1 day; 1	OMT (N = 18)	Sham (N = 17)	**OMT**: Seven standardized techniques (Soft Tissue, Rib Raising, “Redoming” the Abdominal Diaphragm (Indirect Myofascial Release), Suboccipital Decompression, Thoracic Inlet Myofascial Release, Pectoral Traction, TLP With Activation) + Indirect myofascial release, HVLA manipulation, and muscle energy techniques **Sham**: Light hand touch applied to the same anatomic region as that for the OMT maneuver. Light motion testing was used for tissue direction preference but without using myofascial release	Primary outcomes *PFT (FEV_1_, FVC, FEV_1_/FVC, FEF_25%_, FEF_50%_, FEF_75%_, FEF_25%-75%_, FEF_Max_, FIVC, FIF_50%_, FIF_Max_, ERV, IC, MVV, SVC, TGV, RV, TLC, RV/TLC, Airway resistance, Airway conductance) Secondary outcomes *Adverse effects *Blinding success *Subjective perceptions of the intervention used
Noll et al. (2009) [55] US	Crossover RCT	Stable elderly COPD GOLD stage: unknown	1 day (4 week wash-out period); 1	OMT (TLP without activation) (N = 24) OMT (TLP with activation) (N = 24) OMT (Myofascial release) (N = 23) OMT (Rib raising) (N = 22)	Minimal-touch (N = 24)	**TLP without activation**: Pressures applied in the pectoral region during exhalation several times and slow removal of hands during inhalation **TLP with activation**: Pressures applied in the pectoral region during exhalation several times and brisk removal of hands during inhalation **Myofascial release**: Releasing myofascial restriction or asymmetry of the diaphragm, thoracic inlet, rib cage, cervical region **Rib raising**: Anterior-posterior mobilization of ribs in a supine position **Minimal-touch**: Deep breathing with auscultation of the lungs and heart, empathetic discussion for the patients’ heart	*PFT (FVC, FEV_1_, FEV_1_/FVC, FEF_25-75%_, FEF_Max_, MVV, SVC, IC, ERV, TGV, RV, TLC, RV/TLC, airway resistance) *Adverse effects *Subjective perception of the treatment
Maskey-Warzechowska et al. (2019) [56] Poland	Crossover RCT	Stable COPD GOLD stage: severe-to-very severe	1 day (2 week wash-out period); 1	OMT (N = 19)	Sham (N = 19)	**OMT**: Suboccipital decompression, deep cervical fascial release, thoracic lymphatic pump, and diaphragm "stretching", which were adapted from Noll et al. (2008) [54] **Sham**: Shoulder joint mobilization by gliding techniques (glenohumeral anterior, posterior and inferior glide, circumduction) and post-isometric relaxation of the shoulder rotators and biceps brachii, which were adapted from Noll et al. (2008) [54]	Primary outcomes *PFT (RV) *Dyspnea (VAS) Secondary outcomes *PFT (mainly RV, FEV_1_, FVC, FEV_1_/FVC, TLC, RV/TLC, IC, FRC)
Putt et al. (2008) [57] Australia	Crossover RCT	Stable elderly COPD GOLD stage: unknown	2 day (3 day wash-out period); 2	Hold and relax (N = 10)	Sham (N = 10)	**Hold and relax**: The subject’s arm was moved passively three times throughout a resistance-free ROM using glenohumeral flexion and extension. Then the subject was asked to try to bend the elbow to meet the resistance applied by the performer at the mid-ROM **Sham**: Light hand touch applied to the same anatomic region as that in the OMT maneuver	Primary outcomes *VC Secondary outcomes *Perceived dyspnea *ACE and XCE *Upper-limb ROM (both shoulder) *Respiratory rate
Zanotti et al. (2012) [58] Italy	RCT	Stable elderly COPD GOLD stage: severe	4 weeks; 4	OMT + PR (N = 10)	Soft manipulation + PR (N = 10)	**OMT**: Anamnesis, physical examination of the thoracic outlet, spine, rib cage, and thoracic and pelvic diaphragm, and cranial and craniosacral evaluation, with treatment of joint restrictions (not described in detail, It seems to have used various techniques) **Soft manipulation**: Not described **PR**: Exercise training (cycle, cycle ergometer), educational support, nutritional intervention, and psychological counseling	Primary outcomes *6MWT Secondary outcomes *PFT (FEV_1_, FVC, VC, RV)
Kurzaj et al. (2013) [59] Poland	RCT	Unstable COPD GOLD stage: moderate-to-severe	6 days; 6	Specialized Physiotherapy + Standard therapy (N = 20)	Standard therapy (N = 10)	**Specialized Physiotherapy**: A series of six additional massage treatments, which consisted of stroking, grinding, vibration, and kneading techniques **Standard therapy**: Standard pharmacological treatment along with basic physiotherapy. The basic physiotherapy consists of chest relaxation exercises, abdominal exercises combined with prolonged exhalation, active exercises of peripheral joints, walks along the corridor—150 m/day	*BODE index, which consists of four categories 1. BMI 2. Obstruction: FEV_1_ 3. Dyspnea: MRC scale 4. Exertion: 6MWT
Rocha et al. (2015) [60] Brazil	RCT	Stable elderly COPD GOLD stage: severe	2 weeks; 6	Manual diaphragm release technique (N = 10)	Sham (N = 9)	**Manual diaphragm release technique**: Therapist makes manual contact on the underside of the 7^th^ to 10^th^ rib. The therapist gently pulls contacted points in the direction of the head and slightly laterally during inspiration. During exhalation, the therapist progressively deepens contact. Maneuver performed in two sets of 10 breaths with a 1-minute interval between them **Sham**: Same maneuvers executed with light hand touch without any pressure or traction	Primary outcomes *Diaphragmatic mobility Secondary outcomes *6MWT *Maximal respiratory pressures *Abdominal and chest wall kinematics
Buscemi et al. (2019) [61] Italy	RCT	Stable COPD GOLD stage: moderate-to-severe	8 weeks; 8	OMT + Conventional pharmacological therapy (N = 36^¶^)	Conventional pharmacological therapy (N = 36^¶^)	**OMT**: Myofascial release techniques for the treatment of maxillary sinus, vertebral-pleural ligaments, phrenic nerves, ribs, pleura, lungs, bronchi, subclavian muscles, and trapezoid and conoid ligaments **Conventional pharmacological therapy**: once-daily indacaterol-glycopyrronium 25mg/43mg	*CAT *6MWT *PFT (FEV_1_,FVC)
Shakil-ur-Rehman et al. (2013) [62] Pakistan	RCT	COPD GOLD stage: unknown	3 weeks; 15	Rib cage mobilization (N = 35)	Deep breathing exercise (N = 27)	**Rib cage mobilization**: Three techniques; For technique Ⅰ for the left 10^th^ through the 6^th^ ribs, the operator brings the ribcage into right side using the proximal humerus during inspiration; For technique Ⅱ for the right 10^th^ through the 2^nd^ ribs, the operator holds back the lower rib and pulls the upper ribs cranially with inspiration; For technique Ⅲ for the 1^st^ rib, the operator presses the 1^st^ rib downward, medially, and anteriorly **Deep breathing exercise**: Performed by the patient in a relaxed and comfortable position, including the supine position and long supported sitting	*PFT (FEV_1_/FVC ratio)
Engel et al. (2016) [63] Australia	RCT	Elderly COPD GOLD stage: moderate-to-severe	8 weeks; 16	ST + PR (N = 8 at 16 weeks) ST + SM + PR (N = 8 at 16 weeks)	PR (N = 15 at 16 weeks)	**ST**: Massage consisted of gentle effleurage, friction, and cross-fiber friction applied to the muscles of the posterior chest wall, including the intercostal, serratus posterior and anterior, rhomboid, trapezius, latissimus dorsi, erector spinae, quadratus lumborum, and levator scapulae muscles **SM**: The graded delivery of HVLA joint manipulation to the thoracic inter-vertebral, costovertebral, and costotransverse joints **PR**: A 24-week program made up of intervention and non-intervention phases (an 8-week ‘Introductory’ stage, an 8-week ‘Maintenance’ stage, and then an 8-week non-intervention phase)	PFT (FEV_1_, FVC) 6MWT SGRQ HAD scale systolic and diastolic blood pressure
Wada et al. (2016) [64] Brazil	RCT	Stable elderly COPD GOLD stage: moderate-to-severe	12 weeks; 24	Respiratory muscle stretching (hold-relax and passive stretching) + EX (N = 14)	Sham + EX (N = 14)	**Stretching**: Passive elongation of muscle followed by isometric contraction of scalene, sternocleidomastoid, trapezius, pectoralis major and minor, intercostal, serratus anterior, and rectus abdominis muscles. 3 times with 1-min rest between repetitions, before aerobic training **Sham**: Active stretching of wrist and ankle flexors and extensors, contraction held for 1 min with 1 min of rest **EX**: Aerobic training on a treadmill at 60% of average speed achieved during 6MWT, with progression up to 85%	Primary outcomes *6MWT *Dyspnea (a modified Borg scale) *Thoracoabdominal kinematics (optoelectronic plethysmography) Secondary outcomes *Lung function (not described) *Respiratory muscle activity (surface electromyography)
Chen Q et al. (2006) [65] China	RCT	Stable elderly COPD GOLD stage: moderate-to-severe	8 weeks; 40	Chuna + Routine pharmacologic therapy (N = 15)	Routine pharmacologic therapy (N = 15)	**Chuna**: Complex manipulation (grasping and pushing on the head and neck; scrubbing the chest, shoulder, lumbar, hypochondrium; scrubbing, grasping, rotating, and shaking the upper arm; vibrating the acupoints) **Routine pharmacologic therapy**: Oral or inhaled short-acting β_2_ agonists, antitussive and expectorant drugs	*PFT (FEV_1_, FVC, FEV_1_/FVC) *6MWT *mMRC dyspnea scale
Miller WD(1975) [66] US	RCT	COPD GOLD stage: unknown	Unknown	OMT + Routine treatment (N = 13)	Routine treatment (N = 10)	**OMT**: Techniques to hyperextend the dorsal spine using several techniques. Others to increase any restrictive motion. Another to increase lymphatic flow by applying pressure to the muscles of the thoracic cage through anterior compression of the chest. **Routine treatment**: The same appropriate chemical, medical, and adjunctive therapy, including bronchodilators, aerosol, intermittent positive pressure breathing, breathing exercises, postural drainage graded exercises, and supplemental oxygen inhalation	*Neuromusculoskeletal dysfunction *Arterial blood gases and pH (pH, PO_2_, PCO_2_) *Carbon monoxide diffusion studies (DL_COss_, Tidal volume, Minute ventilation) *PFT (VC, Functional residual capacity, RV, TLC, RV/TLC, FEV_1.0_, FEV_2.0_, FEFR, MVV)

**Abbreviations**: COPD, chronic obstructive pulmonary disease; OMT, Osteopathic manual therapy; HVLA, High-velocity low-amplitude; ST, soft tissue therapy; SM, spinal manipulative therapy; PR, Pulmonary rehabilitation; TLP, Thoracic lymphatic pump; EX, Exercise; PFT, Pulmonary function test; FVC, forced vital capacity; FEV_1_, forced expiratory volume in 1 second; 6MWT, 6-minute walking test; mMRC, modified Medical Research Council; FEF_25-75%_, average forced expiratory flow rate over the middle 50% of the FVC; FEFmax, maximum forced expiratory flow rate; MVV, maximal voluntary volume; SVC, slow vital capacity; IC, inspiratory capacity; ERV, expiratory reserve volume; TGV, total gas volume; RV, residual volume; TLC, total lung capacity; VC, vital capacity; FEFR, forced expiratory flow rate; FRC, functional residual capacity; CAT, COPD assessment test; ACE, axillary chest expansion; XCE, xiphisternal chest expansion; ROM, range of motion; SGRQ, St. George’s Respiratory Questionnaire; HAD scale, Hospital Anxiety and Depression Scale.

^¶^total sample size. the number of each group was not reported.

A total of 394 patients were assessed across the 13 studies, and 9 of the RCTs recruited outpatients [54–58, 60, 62, 64, 65]. The age of the participants ranged from 57 to 71 years. No study evaluated the syndrome/pattern differentiation for symptoms and signs in patients with COPD based on traditional Korean and/or Chinese medicine (TKM and TCM, respectively).

Based on the GOLD report, five studies recruited individuals with the moderate-to-severe stage of COPD [59, 61, 63–65], and three studies recruited those with a severe stage of COPD [56, 58, 60]. In the other five studies, severity was classified as “unknown” since the severity of COPD could not be determined due to insufficient baseline characteristics [54, 55, 57, 62, 66].

The sample size in the intervention and control groups ranged from 9 to 35 individuals, with an average of 16.5 (SD, 6.7) individuals, and the treatment periods ranged from 1 to 112 days, with an average of 36.5 (SD, 36.3) days. The total treatment duration ranged from 1 day to 16 weeks, including the wash-out period [55], and from 1 day to 12 weeks, excluding the wash-out period [64]. The total number of treatments ranged from 1 to 40 sessions, with an average of 10.7 (SD, 11.6) sessions. The treatment frequency for multiple-session treatments ranged from 1 to 6 sessions per week [58–65]. The therapies investigated in the included trials were osteopathic techniques [54–56, 58, 61, 66], Chuna [65], soft tissue therapy [63], spinal manipulative therapy [63], specialized physiotherapy [59], hold and relax [57, 64], manual diaphragm release [60], and rib cage mobilization [62]. The interventions were performed by physicians [65], osteopathic physicians [54–56, 58, 61], and physiotherapists [57, 60, 63, 64], all of whom were certified therapists.

The analyses results for the articles included in the study designs are as follows:

Group 1 (G1). MT *versus* Sham [54–57, 60]
Group 2 (G2). MT + CT *versus* CT alone [61, 65]
Group 3 (G3). MT *versus* Deep breathing exercise [62]
Group 4 (G4). MT + PR *versus* sham + PR [58, 64]
Group 5 (G5). MT + PR *versus* PR alone [59, 63, 66]

### Study quality and risk of bias

**Fig 2:**
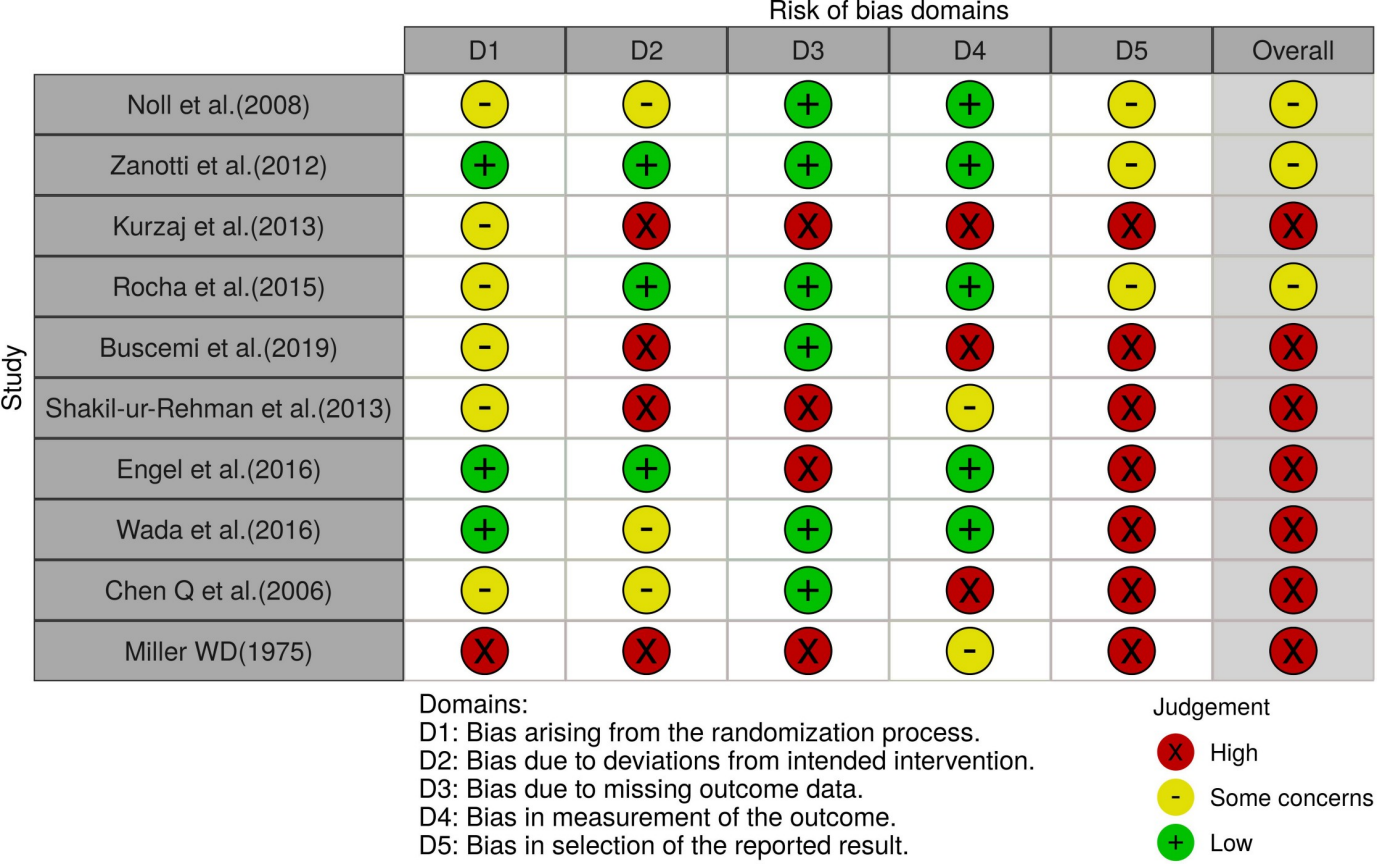
Summary of risk of bias based on analysis of the crossover trials using the Cochrane RoB 2.0 tool.

RoB 2.0 was used to evaluate the effect of assigned intervention for the three crossover trials [55–57]. The results of the RoB analysis are presented in Fig 2 and the S3 File. Additionally, three studies [55–57] showed a low risk of bias for "measurement of the outcome" and “selection of the reported result” categories. The overall biases of the crossover studies were high [57], some concern [55], and low [56], because of the influence of randomization (domain 1) and performance biases (domain 2).

**Fig 3:**
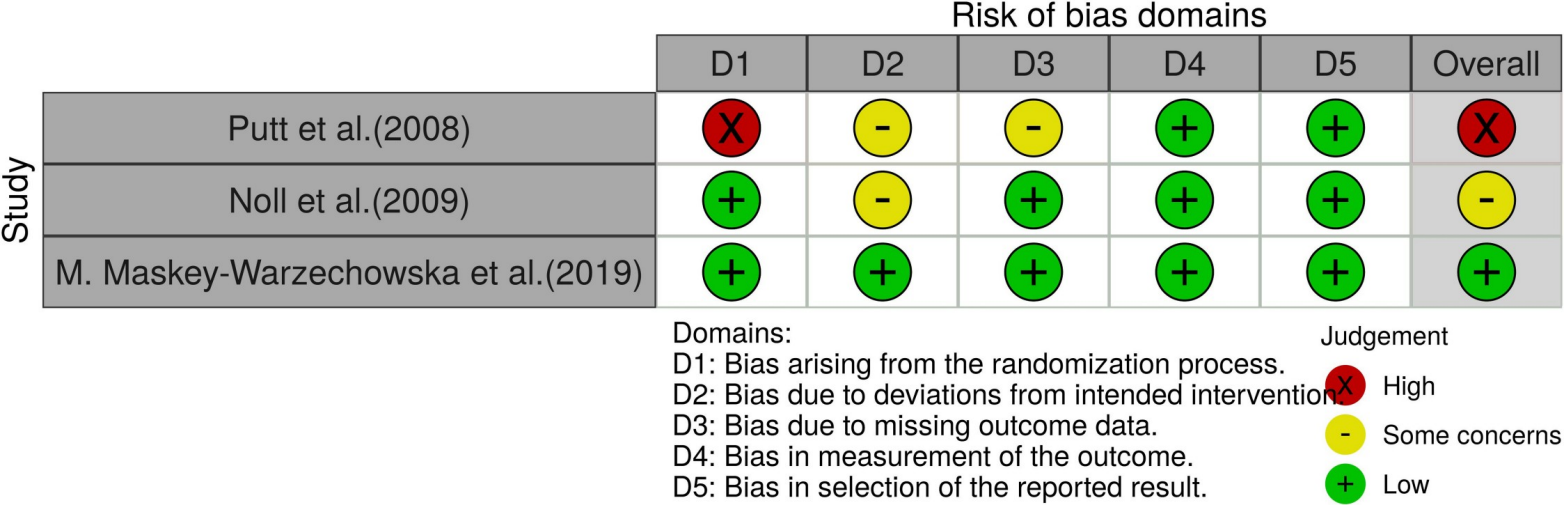
Summary of risk of bias based on the analysis results using the Cochrane RoB 2.0 tool for individually randomized parallel-group trials.

ROB 2.0 was used to evaluate the effect of assigned intervention for 10 studies [54, 58–66]. The results of the RoB analysis are presented in Fig 3 and the S4 File. Four studies mentioned randomization without describing the detailed methods of allocation and/or concealment [54, 59, 61, 65]. Of the studies, seven did not have adequate blinding between participants and healthcare providers [54, 59, 61, 62, 64–66]. In the domain of selection of the reported result, no study had a low risk of bias. Therefore, regarding the overall bias, no study had a low risk of bias.

### Outcomes

By considering the heterogeneity of the studies, a separate analysis was performed on each outcome depending on the study design. If the same variable was observed in each study design, a quantitative analysis was performed. Otherwise, a qualitative description was provided. The main outcomes of this review are summarized in Tables 2 and 3.

**Table 2:** Summary of primary outcomes.

Reference	FEV1 (L)	FVC (L)	FEV1/FVC (%)	VC (L)	RV (L)	TLC (L)	Extra PFT	6MWT (m)
	(Before (B) and After (A))	(Before (B) and After (A))	(Before (B) and After (A))	(Before (B) and After (A))	(Before (B) and After (A))	(Before (B) and After (A))	items	(Before (B) and After (A))
Noll et al. (2008) [54] US	Treatment (B) 1.22±0.65 (A) 1.18±0.62 Control (B) 1.26±0.57 (A) 1.28±0.63	Treatment (B) 2.50±0.94 (A) 2.36±0.93 Control (B) 2.71±0.87 (A) 2.66±0.92	Treatment (B) 47.72±13.23 (A) 48.89±12.88 Control (B) 46.41±13.05 (A) 45.88±16.66	None	Treatment (B) 4.37±2.09 (A) 5.02±3.06↑ ** Control (B) 5.03±1.68 (A) 4.84±1.84	Treatment (B) 6.75±2.02 (A) 7.25±2.91↑ ** Control (B) 7.62±2.01 (A) 7.34±1.98	FEF_25%_, FEF_50%_, FEF_75%_, FEF_25%-75%_, FEF_MAX_, FIVC, FIF_50%_, FIF_MAX_, ERV, IC, MVV, SVC, TGV, RV/TLC, Airway resistance, Airway conductance	None
Noll et al. (2009) [55] US	Minimal-touch control (B) 1.57±0.79 (A) 1.57±0.79 TLP with activation (B) 1.59±0.82 (A) 1.58±0.81 TLP without activation (B) 1.63±0.78 (A) 1.59±0.75 Rib raising (B) 1.51±0.79 (A) 1.53±0.82 Myofascial release (B) 1.56±0.75 (A) 1.52±0.71↓ **	Minimal-touch control (B) 2.79±0.99 (A) 2.80±0.97 TLP with activation (B) 2.83±1.05 (A) 2.79±1.00↓ ** TLP without activation (B) 2.91±1.05 (A) 2.85±1.01 Rib raising (B) 2.75±1.02 (A) 2.77±1.05 Myofascial release (B) 2.83±1.02 (A) 2.79±0.97	Minimal-touch control (B) 55±13 (A) 54±13 TLP with activation (B) 54±13 (A) 55±14 TLP without activation (B) 55±13 (A) 55±13 Rib raising (B) 53±12 (A) 54±13 Myofascial release (B) 54±12 (A) 53±11	None	Minimal-touch control (B) 3.36±0.81 (A) 3.19±0.84 TLP with activation (B) 3.30±0.77 (A) 3.41±0.93↑ ** TLP without activation (B) 3.38±0.92 (A) 3.33±0.99 Rib raising (B) 3.50±1.21 (A) 3.37±1.01 Myofascial release (B) 3.41±0.96 (A) 3.48±1.08	Minimal-touch control (B) 6.27±1.16 (A) 6.10±1.03 TLP with activation (B) 6.27±1.14 (A) 6.29±0.99 TLP without activation (B) 6.41±1.11 (A) 6.33±1.21 Rib raising (B) 6.32±1.31 (A) 6.21±1.14 Myofascial release (B) 6.47±1.23 (A) 6.44±1.27	FEF_25-75%_, FEF_MAX_, MVV, SVC, IC, ERV, TGV, RV/TLC, airways resistance	None
Maskey-Warzechows et al. (2019) [56] Poland	Results presented as median (IQR) Treatment (B) 1.1 (0.8–1.4) (A) 1.0 (0.7–1.3) Control (B) 1.0 (0.7–1.3) (A) 1.0 (0.8–1.3)	Results presented as median (IQR) Treatment (B) 2.9 (2.4–3.7) (A) 3.2 (2.2–3.7) Control (B) 3.0 (2.4–3.6) (A) 2.9 (2.3–3.7)	Results presented as median (IQR) Treatment (B) 33.3 (29.4–43.1) (A) 33.2 (30.0–43.3) Control (B) 32.4 (29.2–43.1) (A) 31.4 (28.5–43.7)	None	Results presented as median (IQR) Treatment (B) 4.5 (3.8–4.9) (A) 4.5 (3.8–4.8) Control (B) 4.5 (4.2–5.2) (A) 4.5 (4.1–5.1)	Results presented as median (IQR) Treatment (B) 7.5 (6.5–9.0) (A) 7.5 (6.6–8.7) Control (B) 7.6 (6.9–9.0) (A) 7.2 (6.5–8.8)	FEV1% prd, FVC% prd, TLC% prd, RV% prd, Airway resistance, IC, FRC, FRC% of prd	None
Putt et al. (2008) [57] Australia	None	None	None	Day 1 Treatment (b) 3.06±0.6 (a) 3.5±0.5↑ * Sham (b) 3.4±0.5 (a) 3.4±0.5 Day 2 Treatment (b) 3.5±0.6 (a) 3.7±0.6↑ * Sham (b) 3.5±0.5 (A) 3.3±0.5	None	None	None	None
Zanotti et al. (2012) [58] Italy	Treatment (B) 0.99±0.4 (A) 1.13±0.4 Control (B) 0.89±0.4 (A) 0.90±0.4	Treatment (B) 1.96±0.7 (A) 2.05±0.6 Control (B) 1.75±0.7 (A) 1.79±0.8	None	Treatment (B) 1.76±0.4 (A) 1.87±0.3 Control (B) 1.88±0.8 (A) 1.86±1.0	Treatment (B) 4.4±1.5 (A) 3.9±1.7↓ *** Control (B) 4.29±1.5 (A) 4.23±1.4	None	None	Treatment (B) 297.0±59.3 (A) 369.5±80.0↑ ** Control (B) 281.0±97.4 (A) 304.7±96.6
Kurzaj et al. (2013) [59] Poland	Treatment (B) 1.1±0.19 (A) 1.4±0.26† Control (B) 1.2±0.8 (A) 1.4±0.7‡	None	None	None	None	None	None	Treatment (B) 241.0±78.8 (A) 318.8±73.6 Control (B) 229.0±87.1 (A) 262.5±89.9
Rocha et al. (2015) [60] Brazil	None	None	None	Pre1:Post1 6 treatments (B) 2.00±0.29 (A) 2.14±0.2 Control (B) 2.27±0.39 (A) 2.12±0.38 Pre6:Post6 treatment (B) 2.21±0.38 (A) 2.31±0.36 Control (B) 2.26±0.47 (A) 2.10±0.40	None	None	IC	Pre1:Pre6 (changes after 5 treatment) treatment (B) 446.61±81.20 (A) 461.73±82.47↑ **(statistically significant between-group difference, but *P*-value not reported)** Control (B) 421.56±63.01 (A) 415.11±61.74
Buscemi et al. (2019) [61] Italy	*4 weeks (on the same day as the 4th OMT session, T3) Spirometry improved but was not statistically different between groups (*P* < 0.5061)	*4 weeks (on the same day as the 4th OMT session, T3) Spirometry improved but was not statistically different between groups (*P* < 0.5411)	None	None	None	None	None	*4 weeks (on the same day as the 4th OMT session, T3) Pre- and post-treatment 6MWD improved in a statistically significant in the group (Treatment: *P* < 0.0038; Control: *P* < 0.5326) *10 weeks (15 days after the last OMT session, T6) Pre- and post-treatment 6MWD improved in a statistically significant in the group (Treatment: *P* < 0.05; Control: *P*-values not reported)
Shakil-ur-Rehman et al. (2013) [62] Pakistan	None	None	Not described in detail. "The results of rib cage mobilization in group A were statistically significant compared to those of deep breathing exercises in group B." without data and *P* values.	None	None	None	None	None
Engel et al. (2016) [63] Australia	No raw data(L) 16 weeks (mean change with 95% CI) ST + PR -0.021 (-0.115, 0.072) ST + SM + PR -0.020 (-0.136, 0.096) PR -0.042 (-0.113, 0.029) 24 weeks (mean change with 95% CI) ST + PR -0.089 (-0.175, -0.003) ST + SM + PR -0.020 (-0.144, 0.104) PR -0.077 (-0.164, 0.011)	A significant difference was noted among the three groups at 24 weeks **. No raw data (L) 16 weeks (mean change with 95% CI) ST + PR 0.45 (0.13, 0.77) ST + SM + PR 0.37 (0.22, 0.53) PR 0.10 (-0.14, 0.35) 24 weeks (mean change with 95% CI) ST + PR 0.32 (-0.05, 0.68) ST + SM + PR 0.53 (0.26, 0.81) (*P* = 0.04 between groups) PR 0.10 (-0.14, 0.34)	None	None	None	None	None	A significant difference among the three groups was noted at 16 weeks ** and 24 weeks **. No raw data (m) 16 weeks (mean change with 95% CI) ST + PR 5.8 (-25.1, 36.7) ST + SM + PR 51.7 (29.8, 73.6) PR 22.7 (-6.1, 51.4) 24 weeks (mean change with 95% CI) ST + PR -16.4 (-55.1, 22.2) ST + SM + PR 35.0 (-1.5, 71.5) PR 12.1 (-18.0, 42.2)
Wada et al. (2016) [64] Brazil	None	None	Only baseline scores were reported. Treatment (b) 0.56±0.13 Control (b) 0.49±0.09	None	None	None	None	Treatment (B) 473±68 (A) 488.0±17.4↑ *** Control (B) 439±103 (A) 454±17.5
Chen Q et al. (2006) [65] China	Treatment (B) 1.248±0.743 (A) 1.419±0.953↑ ** Control (B) 1.269±0.881 (A) 1.333±0.798	Treatment (B) 2.311±0.875 (A) 2.628±0.921↑ ** Control (B) 2.266±0.956 (A) 2.362±0.759	Treatment (B) 47.63±10.69 (A) 54.57±11.25↑ ** Control (B) 48.25±11.71 (A) 50.60±9.62	None	None	None	None	Treatment (B) 330.51±67.21 (A) 389.73±72.15↑ ** Control (B) 328.79±71.13 (A) 346.65±69.23
Miller WD (1975) [66] US	Treatment (B) 72.4±3.5 (A) 74.5±2.9 Control (B) 77.6±3.3 (A) 75.2±3.0	None	None	Treatment (b) 2.3±0.2 (a) 2.8±0.2 Control (b) 2.4±0.2 (a) 2.5±0.2	Treatment (b) 1.9±0.2 (a) 2.4±0.2 Control (b) 2.0±0.2 (a) 2.0±0.3	Treatment (b) 4.1±0.4 (a) 5.1±0.3 Control (b) 4.4±0.4 (a) 4.5±0.4	Carbon monoxide diffusion studies, FEV_2.0_, FEFR	None

The arrows signify statistically significant differences either within or between groups with *P* values. Most studies measured outcomes before and after treatment. Only in Engel’s study, the mean change value is given instead of before- and after-treatment values.

*Significantly different between groups at *P*<0.01.

**Significantly different between groups at *P*<0.05.

***Significantly different between groups at *P*<0.001.

†Significantly different in the group at *P* = 0.0001.

‡Significantly different in the group at *P* = 0.0050.

**Abbreviations**: FVC, forced vital capacity; FEV_1_, forced expiratory volume in 1 second; FEF_25-75%_, average forced expiratory flow rate over the middle 50% of the FVC; FEF_MAX_, maximum forced expiratory flow rate; MVV, maximal voluntary volume; SVC, slow vital capacity; IC, inspiratory capacity; ERV, expiratory reserve volume; TGV, total gas volume; RV, residual volume; TLC, total lung capacity; VC, vital capacity; FEFR, forced expiratory flow rate; TLP, Thoracic lymphatic pump; FRC, functional residual capacity.

**Table 3:** Summary of secondary outcomes.

Reference	Symptoms results	Quality of life	Adverse events	Follow-up
Noll et al. (2008) [54] US	Patient-reported outcomes *Rate of breathing better: "Yes:No" 14:3 treatment 11:5:1 control (Yes:No:Uncertain)	None	Patient-reported outcomes *Having any adverse side effects: "Yes:No" 2:15 treatment 4:13 control No major muscle soreness, elevated blood pressure, heart palpitations	No
Noll et al. (2009) [55] US	Patient-reported outcomes *Rate of breathing better: "Yes:No" 8:10 Minimal-touch control 17:6 TLP with activation 12:9 TLP without activation 15:4 Rib raising 8:8 Myofascial release	None	Patient-reported outcomes Rate: "side effect/total number of surveyed patients for each techniques" 1/18 Minimal-touch control 4/23 TLP with activation 4/21 TLP without activation 3/20 Rib raising 2/16 Myofascial release Chest pain, soreness in the front chest, cramps in the left lung, discomfort across the back, stiff neck, headache	No
Maskey-Warzechowska et al. (2019) [56] Poland	Dyspnea VAS, Results presented as median (IQR), (Before: After) Treatment 3.0 (0.5–6.0): 3.0 (1.0–4.5) Control 4.0 (1.0–5.5): 2.0 (1.0–4.0)	None	0 “No adverse effects associated with the OMT and sham intervention were observed in any of the participating patients.”	No
Putt et al. (2008) [57] Australia	Borg scale of dyspnea [median(range)] (Before: After) Day 1 treatment 1(0–3):1(0–2) Day 2 treatment 1(0–3):1(0–3) Day 1 sham 1(0–3):1(0–3) Day 2 sham 1(0–3):1(0–3)	None	None	No
Zanotti et al. (2012) [58] Italy	a modified Borg scale (at the end of 6MWT) "Moreover, patients treated with OMT reported subjective improvement in their breathing"	None	0 “There were no adverse effects or side effects.”	No
Kurzaj et al. (2013) [59] Poland	the MRC scale [0–3 points] (Before: After) treatment (B) 2.10±0.77 (A) 1.20±0.83 control (B) 1.7±0.7 (A) 1.4±0.5	None	None	No
Rocha et al. (2015) [60] Brazil	None	None	None	No
Buscemi et al. (2019) [61] Italy	The scale of dyspnea and fatigue was not described. *4 weeks (on the same day as the 4th OMT session, T3) The pre- and post-intervention dyspnea improved in a statistically significant in the treatment group (*P*-values not reported), while there were no changes for the fatigue parameter for either group. *10 weeks (15 days after the last OMT session, T6) The pre- and post-intervention dyspnea improved in a statistically relevant way in the treatment group (*P*-values not reported), while there were no changes for the fatigue parameter for either group.	CAT *4 weeks (on the same day of the 4th OMT sessions, T3) Pre-post intervention CAT scores improved in the treatment group (Treatment: *P* < 0.0005, Controls: *P* < 0.188). *10 weeks (15 days after the last OMT session, T6) Pre- and post-intervention CAT scores improved in the treatment group (Treatment: *P* < 0.05, Controls: *P*-values not reported).	“No adverse events were recorded”; “only during the first three sessions some subjects reported an increase in the amount of mucus and pain in the maxillary bone.”	Yes 15 days after the last OMT session
Shakil-ur-Rehman et al. (2013) [62] Pakistan	None	None	None	No
Engel et al. (2016) [63] Australia	None	SGRQ In all three groups, SGRQ scores decreased after 16 and 24 weeks (especially in the two groups PR and ST+SM+PR), but there was no significant difference	Patient-reported outcomes Two mild AEs (muscle soreness) were reported by participants in the ST+PR group. No major or moderate AEs	Yes Checked effects 4 weeks, 12 weeks after treatment (medium effects)
Wada et al. (2016) [64] Brazil	Modified Borg scale [0–10 points] dyspnea after the 6MWT (scores before 6MWT were not described) treatment 1.53±0.31**↓** ** control 2.78±0.30	None	None	No
Chen Q et al. (2006) [65] China	Cured/markedly progress/progress/invalid (total effective rate)† treatment: 2/8/3/2(67%) **↑** * control: 0/6/6/3(40%)	None	None	No
Miller WD (1975) [66] US	None	None	None	No

The arrows signify statistically significant differences within or between groups with *P* values.

*Significantly different between groups at the *P*<0.01.

**Significantly different between groups at the *P*<0.001.

†Judgment criteria for dyspnea—Cured: symptoms of dyspnea disappeared; marked progress: symptoms of dyspnea were significantly reduced (more than two levels in mMRC dyspnea scale); progress: symptoms of dyspnea were alleviated (within one level); invalid: no improvement in dyspnea symptoms.

**Abbreviation**: COPD, chronic obstructive pulmonary disease; TLP, thoracic lymphatic pump; SGRQ, the St. George’s Respiratory Questionnaire; mMRC scale, modified Medical Research Council scale; MRC scale, Medical Research Council scale; AE, adverse events; VAS, visual analogue scale; CAT, COPD assessment test.

#### G1. MT *versus* sham

The effects of MT compared to sham treatments were reported in five trials [54–57, 60], and the short-term effects of 1–2 treatment on lung function were evaluated in four studies [54–57].

*Primary outcomes (PFT*, *6MWD)*. In terms of primary outcomes, a meta-analysis could only be performed for VC among PFT parameters. FEV_1,_ FVC, FEV_1_/FVC, RV, and TLC were all reported on in three articles, with only one treatment [54–56].

**Fig 4:**
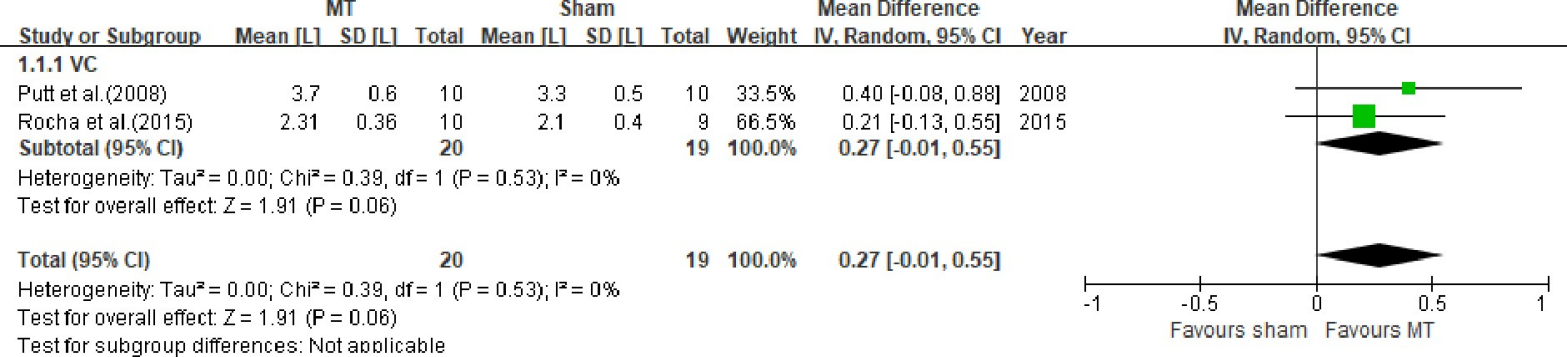
Forest plots for comparison of vital capacity (VC) between manual therapy (MT) and sham.

(1) PFT

1) FEV_1_: FEV_1_ decreased by 3.3% (-0.04 L, SD 0.636) after the intervention compared to the pre-intervention value (mean±SD, 1.22±0.65 L) in the integrative osteopathic MT (OMT) intervention group, which was not a significant difference [54]. This was also the case in a study using the same integrative OMT [56]. Myofascial release also resulted in a decrease in FEV_1_ by 2.6% (*P* = 0.03) after a single OMT intervention [55].

2) FVC: FVC decreased by 5.6% (-0.14 L, SD 0.935) after the intervention compared to the pre-intervention value (mean±SD, 2.50±0.94 L) in the integrative OMT intervention group, which was not a significant difference [54]. However, in the study using the same integrative OMT [56], FVC increased by 0.3 L in the intervention group, which was also not statistically different. Thoracic lymphatic pump (TLP) with activation also decreased FVC by 4.9% after a single OMT intervention (*P*<0.05) [55].

3) FEV_1_/FVC: FEV_1_/FVC(%) increased by 2.5% (1.17, SD 13.059) in the integrative OMT intervention group, which was not a significant difference [54]. In a study using the same integrative OMT [56], FEV_1_/FVC decreased by 0.1% in the intervention group, which was also not statistically different. There was no significant change in FEV_1_/FVC(%) after a single OMT intervention [55].

4) VC: Through the meta-analysis, we found that VC did not improve after two [57] and six interventions (60). Fig 4 summarizes these results (for 57, 60; MD 0.27, 95% CI -0.01 to 0.55, I^2^ = 0%).

5) RV: RV increased by 14.9% (0.65 L, SD 2.709) in the integrative OMT intervention group (*P*<0.05) [54]. TLP with activation also increased RV by 3.3% (*P*<0.05) [55]. However, in another study using the same integrative OMT, RV showed no significant change [56].

6) TLC: TLC increased by 7.4% (0.5 L, SD 2.583) in the integrative OMT intervention group (*P*<0.05) [54]. However, there was no significant difference in TLC values between the experimental and control groups in other studies [55, 56].

(2) 6MWD

Only one study by Putt et al. [57] reported 6MWD findings. In this study, the 6MWD score increased after performing hold and relax five times (15.12 m, 95% CI -66.722 to 96.962), whereas it decreased in the control group (6.45 m, 95% CI -68.835 to 68.835). The *P*-value was not reported in this study.

*Secondary outcomes (symptoms*, *QoL*, *AE)*. (1) Symptoms: Dichotomous questions were used in two studies to describe the results of patient-perceived symptomatic improvements after OMT [54, 55], and one study reported perceived dyspnea using the Borg scale [57], while yet another study used a VAS for dyspnea [56]. The percentage reflected for dichotomous results, instead of the odds ratio, due to the multi-arm crossover RCT design. Positive response rates of symptoms after treatments were 82% [54] and 50–78% [55]; however, those of controls were 68% [54] and 44% [55], respectively. No changes were observed in the pre- or post-intervention results for either the Borg scale or dyspnea VAS [56, 57].

(2) QoL: No study in this group reported QoL; hence, the evaluation was impossible.

(3) AE: AEs after one-session treatment were found using patient-reported outcomes in two studies [54, 55]. The following types of musculoskeletal pain were the most frequently reported: muscle soreness (in the treatment-involved regions, such as the chest, back, and neck) and cardiovascular symptoms (elevated blood pressure, palpitation, and headache).

#### G2. MT + CT *versus* CT alone

The combination of MT and CT was compared to CT alone in two studies [61, 65] through a qualitative analysis of patients with moderate-to-severe COPD.

*Primary outcomes (PFT*, *6MWD)*. (1) PFT

A dynamic volume was used for the PFT. After a total of 40 Chuna sessions combined with CT over an eight week period, PFTs (FEV_1_, FVC, and FEV_1_/FVC) significantly improved compared to CT alone (*P*<0.05) [65]. The post-intervention values of FEV_1_, FVC, and FEV_1_/FVC increased by 13.7% (0.171 L, SD 0.867), 13.7% (0.317 L, SD 0.899), and 14.6 points (6.94, SD 10.981), respectively. After four integrative OMT sessions combined with CT over a four week period, PFTs (FEV_1_ and FVC) improved, although without statistical significance (*P* < 0.5061) [61].

(2) 6MWD

In terms of exercise capacity, only routine pharmacologic therapy (CT alone) increased the walking distance by 5.4% (17.86 m, SD 70.199), whereas the addition of MT (Chuna) to the routine pharmacologic therapy increased the walking distance by 17.9% (59.22 m, SD 69.811; *P* < 0.05) [65]. After the integrative OMT intervention, the intervention group’s 6MWD significantly increased at both 4 (*P* < 0.0038) and 10 weeks (*P* < 0.05), but the raw data for these results were not reported [61].

*Secondary outcomes (symptoms*, *QoL*, *AE)*. The mMRC score was recalculated according to its own grading scale for improvement when interpreting the mMRC dyspnea scale data of the study in China [65]. More significant improvement in symptoms was observed in patients who had undergone both routine pharmacologic therapy and MT than in those who had undergone routine pharmacologic therapy alone (P<0.01) [65]. Buscemi et al.’s [61] study showed that dyspnea and fatigue improved in the integrative OMT group, but raw data and *P-values* were not reported.

In a study that evaluated QoL using CAT, the change-from-baseline CAT score significantly improved in the treatment group at 4 weeks (treatment group: *P*< 0.0005; control group: *P* < 0.188). Additionally, this study reported that the change-from-baseline CAT score significantly improved in the treatment group at 10 weeks (*P* < 0.05). However, only *P-values*, no raw data, were not provided [61].

No AE was reported in Buscemi et al.’s [61] study, but after three integrative OMT sessions, there were minor side effects that did not require treatment (e.g., muscle and maxilla pain).

#### G3. MT *versus* deep breathing exercise

The study by Shakil-ur-Rehman et al. [62] was included in this category of interventions. The third category was classified separately since the control intervention group had different characteristics than the sham and PR subgroups.

*Primary outcomes (PFT*, *6MWD)*. The study [62] reported that there was a higher increase in FEV_1_/FVC after rib cage mobilization than that in the control group (*P*-value, raw data, and baseline characteristics were not given).

*Secondary outcomes (symptoms*, *QoL*, *AE)*. Although the dyspnea index was used to assess symptoms, the results were not reported. In this group, QoL and AEs were not studied as outcome variables [62].

#### G4. MT + PR *versus* sham + PR

Two studies were included in this category: one study [64] included patients with moderate-to-severe COPD, while the other [58] included patients with severe COPD. Since the number of studies that reported each outcome variable was small, a qualitative analysis was performed. A meta-analysis was performed only using 6MWD findings from on two studies [58, 64].

*Primary outcomes (PFT*, *6MWD)*. (1) PFT

FEV_1_, FVC, VC, and RV were investigated. After four interventions [58], FEV_1_, FVC, and VC in the OMT plus PR group significantly increased by 14.1% (0.14 L, SD 0.4), 4.6% (0.09 L, SD 0.656), and 6.3% (0.11 L, SD 0.361) respectively; however, the difference was not statically significant. In the same study [58], RV decreased significantly (*P*<0.001) in the intervention group by 11.4% (0.5 L, SD 1.609).

(2) 6MWD

**Fig 5:**
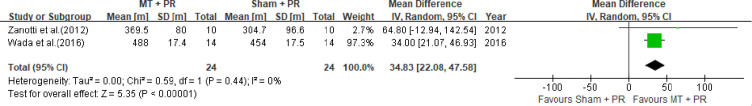
Forest plots for comparison of 6-minute walk distance (6MWD) results between manual therapy (MT) plus pulmonary rehabilitation (PR) and sham plus PR.

The meta-analysis showed that adding MT to exercise treatment or PR has a particularly beneficial effect on 6MWD (for 58, 64; MD 34.83, 95% CI 22.08 to 47.58, I^2^ = 0%; Fig 5)

*Secondary outcomes (symptoms*, *QoL*, *AE)*. When dyspnea was assessed using the modified Borg scale [58, 64], one study [58] described their results narratively without data and *P*-values, and the other study [64] reported that symptoms showed significant improvement based on the difference in mean post-intervention values (*P*<0.001), without presenting the pre-treatment data. No study in this group reported QoL. Only one study [58] reported AEs descriptively, and the number of such AEs was reported as “zero” after treatment once a week for 4 weeks.

#### G5. MT + PR *versus* PR alone

Three studies were included in this category [59, 63, 66]. Two studies [59, 63] conducted clinical research on patients with moderate-to-severe COPD. Miller [66] did not specify the intervention schedule or severity of disease of participants. A study by Engel et al. [63] reported results at 16 and 24 weeks after starting the clinical research. Considering that MT is terminated at the 12^th^ week, we used the results of the 16^th^ week for an analysis of primary outcomes in this review. In addition, this study [63] had limitations in pooling due to its multi-arm RCT research design. Considering that both soft tissue therapy (ST) and spinal manipulative therapy (SM) belong to MT, we combined the results from ST and SM groups and used them for meta-analysis. Using the change-from-baseline value score [63] and post-intervention value score [59, 66], we performed a meta-analysis on FEV_1_ and 6MWD. The remaining outcome variables were analyzed qualitatively.

*Primary outcomes (PFT*, *6MWD)*. (1) PFT

**Fig 6:**
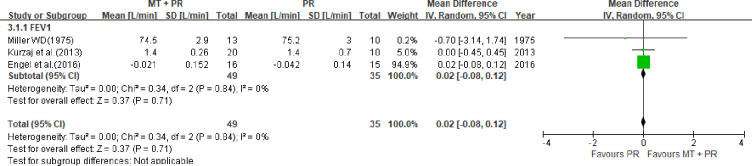
Forest plots for comparison of forced expiratory volume in one second (FEV_1_) between manual therapy (MT) plus pulmonary rehabilitation (PR) and PR alone.

Three studies [59, 63, 66] reported FEV_1_. Fig 6 shows that FEV_1_ did not improve in the MT plus PR group compared to the PR alone group (for 59, 63, 66; MD 0.02, 95% CI -0.08 to 0.12, I^2^ = 0%). In a study by Kurzaj et al. [59], the within-group normal FEV_1_ values improved after the intervention in both the treatment (MT + PR, *P =* 0.0001) and control groups (PR alone, *P* = 0.0050). In a study by Engel [63], FVC improved by 0.385 L (SD 0.363) at 16 weeks in the MT plus PR group. However, the finding was not statistically significant. In a study by Miller [66], VC, TLC, and RV increased by 21.7% (0.5 L, SD 0.2), 24.4% (1.0 L, SD 0.361), and 26.3% (0.5 L, SD 0.2), respectively, none of which were statistically significant.

(2) 6MWD

**Fig 7:**
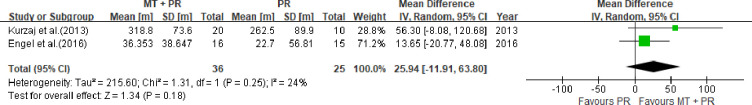
Forest plots for comparison of 6-minute walk distance (6MWD) between manual therapy (MT) plus pulmonary rehabilitation (PR) and PR alone.

A meta-analysis showed that walking distance did not improve in the MT plus PR group compared to that in the PR-only group (for 59, 63; MD 25.94, 95% CI -11.91. 63.80, I^2^ = 24%; Fig 7).

*Secondary outcomes (symptoms*, *QoL*, *AE)*. Only one study [59] reported symptoms in this group. After six interventions of MT plus PR, the MRC scale score decreased further, by 0.9 (SD 0.802); intervention with PR alone decreased the MRC scale score by 0.3 (SD 0.624). However, none of the groups showed a significant difference. One study [63] evaluated QoL using the SGRQ score. In this study [63], all three groups showed a reduction in SGRQ scores after multiple-session treatments, without statistical significance. Only one study [63] documented two mild AEs using patient-reported outcomes after 131 sessions of ST + PR groups, and no moderate or severe AEs were reported after 136 sessions of ST + SM + PR.

### Quality of evidence

The quality of evidence was graded as “very low” to “moderate” (Table 4). No "high" quality of evidence was identified for any parameter, as inconsistencies in the analysis of outcomes, high risk of bias, and small sample sizes were observed in the included RCTs.

**Table 4:** GRADE.

Outcomes		RCTs	Population	ROB	Inconsistency	Indirectness	Imprecision	Publication Bias	GRADE
**Primary outcome**									
**Pulmonary Function Test**									
G1. MT versus Sham	FEV1	3RCTs	78	not serious	not serious	not serious	serious^a^	none	MODERATE
	FVC	3RCTs	78	not serious	not serious	not serious	serious^a^	none	MODERATE
	FEV1/FVC	3RCTs	78	not serious	not serious	not serious	serious^a^	none	MODERATE
	VC	2RCTs	29	serious^b^	serious^c^	not serious	serious^a^	none	VERY LOW
	RV	3RCTs	78	not serious	not serious	not serious	serious^a^	none	MODERATE
	TLC	3RCTs	78	not serious	not serious	not serious	serious^a^	none	MODERATE
G2. MT + CT versus CT alone	FEV1	2RCTs	66	serious^b^	not serious	not serious	serious^a^	none	LOW
	FVC	2RCTs	66	serious^b^	not serious	not serious	serious^a^	none	LOW
	FEV1/FVC	1RCT	30	serious^b^	not serious	not serious	serious^a^	none	LOW
G3. MT versus Deep breathing exercise	FEV1/FVC	1RCT	62	serious^b^	not serious	not serious	serious^a^	none	LOW
G4. MT + PR versus Sham + PR	FEV1	1RCT	20	not serious	not serious	not serious	serious^a^	none	MODERATE
	FVC	1RCT	20	not serious	not serious	not serious	serious^a^	none	MODERATE
	VC	1RCT	20	not serious	not serious	not serious	serious^a^	none	MODERATE
	RV	1RCT	20	not serious	not serious	not serious	serious^a^	none	MODERATE
G5. MT + PR versus PR alone	FEV1	3RCTs	84	serious^b^	serious^c^	not serious	serious^a^	none	VERY LOW
	FVC	1RCT	31	serious^b^	not serious	not serious	serious^a^	none	LOW
	VC	1RCT	23	serious^b^	not serious	not serious	serious^a^	none	LOW
	RV	1RCT	23	serious^b^	not serious	not serious	serious^a^	none	LOW
	TLC	1RCT	23	serious^b^	not serious	not serious	serious^a^	none	LOW
**Six minute walk test**									
G1. MT versus Sham	6WMD	1RCT	10	serious^b^	not serious	not serious	serious^a^	none	LOW
G2. MT + CT versus CT alone	6WMD	2RCTs	66	serious^b^	not serious	not serious	serious^a^	none	LOW
G4. MT + PR versus Sham + PR	6WMD	2RCTS	48	serious^b^	not serious	not serious	serious^a^	none	LOW
G5. MT + PR versus PR alone	6WMD	2RCTS	61	serious^b^	serious^c^	not serious	serious^a^	none	VERY LOW
**Secondary outcome**									
**Symptom**									
G1. MT versus Sham	Effective rate	2RCTS	59	not serious	not serious	not serious	serious^a^	none	MODERATE
	Dyspnea	2RCTs	29	serious^b^	not serious	not serious	serious^a^	none	LOW
G2. MT + CT versus CT alone	Dyspnea	2RCTs	66	serious^b^	not serious	not serious	serious^a^	none	LOW
G4. MT + PR versus Sham + PR	Dyspnea	2RCTs	48	serious^b^	not serious	not serious	serious^a^	none	LOW
G5. MT + PR versus PR alone	Dyspnea	1RCT	30	serious^b^	not serious	not serious	serious^a^	none	LOW
**Quality of life**									
G2. MT + CT versus CT alone	CAT	1RCT	36	serious^b^	not serious	not serious	serious^a^	none	LOW
G5. MT + PR versus PR alone	SGRQ	1RCT	31	serious^b^	not serious	not serious	serious	none	LOW

^a^ studies include relatively few participants.

^b^ based on the results of overall risk of bias.

^c^ unexplained heterogeneity.

## Discussion

### Summary of the systematic review

#### Aims and objectives

The present SR aimed to investigate the efficacy of MT in individuals with COPD. Existing PR has shown advantages in a moderate-to-severe COPD population [17]. Based on these results, we inferred that patients at various stages of COPD may benefit similarly from MT.

#### Summary of results

A comprehensive search found that 13 RCTs were suitable for inclusion in this review [54–66]. We updated five additional studies [56, 59, 61, 62, 65], including one Chinese article, as compared to existing SRs [20–23]. The 13 studies were categorized into 5 subgroups, based on trial design, and the outcomes were analyzed. Study quality was assessed using the Cochrane RoB 2.0.

*The primary outcomes*. (1) G1 and G2: The effects of Short-term MT treatment (G1) could not be determined. Long-term MT treatment (G2) improved FEV_1_, FVC, FEV_1_/FVC, and 6MWD in patients with moderate-to-severe COPD. Compared to the results of G1, MT may be effective for long-term and multi-session treatments.

(2) G4 and G5: Compared to sham plus PR, MT added to PR reduced RV and significantly improved 6MWD in patients with moderate-to-severe COPD (G4). MT added to PR improved FEV_1_ and 6MWD, but the effects were not significant compared to the solely PR group (G5).

*The secondary outcomes*. (1) Dyspnea: The effects of MT on dyspnea were confirmed in four subgroups (G1-2 and G4-5). However, only subgroup G5 did not show a significant difference. The overall effects of MT on symptoms were inconclusive, likely due to heterogeneity in the measurement of symptoms, recalculation of results [65], and incomplete selective reporting [58, 64].

(2) QoL: There is insufficient evidence to state the effects of MT on QoL, although one study reported SGRQ [63] and another reported CAT [61].

(3) AE: The risk ratio of AE increased in the G1 subgroup [54, 55], and two AEs were reported in G5 [63]. However, the AEs described in the present review were mild.

After one MT treatment, FEV_1_ and FVC decreased and RV increased, which was unbeneficial for PFT. This is thought to be due to reduced soft tissue elasticity in older patients, which prevents them from responding to MT [67]. Previous research has recommended extending the treatment period and/or adding PR for these patients [56]. Although the effects of short-term MT were not confirmed (G1), we found that lung function and exercise capacity were improved in patients who had more than eight weeks of MT (G2). MT added to PR significantly improved exercise capacity in subgroup G4, and addressed symptoms of dyspnea in both G4 and G5. Therefore, further research is needed to investigate the effects of MT in improving exercise capacity and alleviating shortness of breath. However, a definite conclusion could not be established in the present review, due to the increased heterogeneity in trial designs and interventions evaluated, as well as the high risk of bias for the available evidence. Clinicians may refer to the present study on whether MT should be used for personalized treatment.

### Assessment of the risk of bias in manual therapy research

The importance of deviations from intended interventions (performance bias) can vary according to trial design. Concerning participant blinding, a stricter judgment must be made in the MT vs. sham-controlled design (G1, G4), whereas the MT vs. non-sham design (G2, G3, G5) requires less strict judgments. Such a difference in the importance of blinding is not reflected in the indiscriminating judgment made by domain two of the RoB 2.0 tool. Among the seven studies belonging to the MT vs. sham control design group (G1, G4), there was no study with a high risk of bias (0/4/3; high/some concerns/low). Among the six studies belonging to the MT group vs. the non-sham design group (G2, G3, G5), there were three studies with a high risk of bias (4/1/1; high/some concerns/low).

### Study limitations

The heterogeneity in the study design of the included trials must be considered in the interpretation of the results. The included studies differed in terms of disease severity of the participants, treatment techniques, length and intensity of treatment, and the total duration of treatment. Moreover, none complied with the Consolidated Standards of Reporting Trials (CONSORT) extension for non-pharmacologic treatment (NPT) [68] for reproducibility and transparency of RCTs [54–66].

### Study strengths

This review provided updated information based on available journal articles that investigated the effect of MT in patients with COPD. Due to the lack of language barriers in East Asian databases, we could include new research on Chuna treatments for COPD [65]. Although the heterogeneity of MT was a significant limitation for researchers, the present review addressed this limitation for the first time by classifying the included articles based on their trial design. MT, accompanied by CT, indicated that multiple-session MT may possibly improve exercise capacity and lung function in patients with COPD. The possibility of alleviating symptoms was also observed when MT was added to either PR or CT. Moreover, the present review has the advantage of evaluating associated AEs, whereas previous SRs did not [20, 22].

### Study implications for practice and further research

To create a high level of evidence regarding the efficacy of MT via SR, future studies that comply with reporting guidelines are required so that the methodological quality can be improved and more robust comparisons of different MT interventions be conducted. Given the inherent challenges of blinding in trials for studying MT [68], pragmatic trial designs may be worth considering. There is a need to recruit clinical trial participants by considering the severity of the disease according to official reports (e.g., GOLD 2020, [17]) for subgroup analysis. Given that QoL is an important measure to analyze the comprehensive health of patients with COPD [69, 70], more research on QoL is needed. Additionally, evidence for the follow-up results of MT is needed, since these were obtained in two studies [61, 63].

## Conclusion

This review showed that there is insufficient evidence to support the role of MT in the management of individuals with COPD. This is because the included studies in each subset were too small, resulting in a smaller number of studies available for meta-analysis. Furthermore, the studies were of poor quality, with some concerns regarding a high risk of bias. In the future, high-quality studies designed to evaluate the effect of MT thoroughly should be conducted. In addition to this review, practitioners need to use clinical judgment for the utilization of MT.

## Supporting information

S1 File PRISMA, 2009 checklist. (DOC)

S2 File Search strategy. (DOCX)

S3 File Evaluation of the risk of bias based on the Cochrane RoB 2.0 tool for crossover trials. (XLSM)

S4 File Evaluation of the risk of bias based on the Cochrane RoB 2.0 tool for individually randomized parallel-group trials. (XLSM)
